# A unique Levey–Jennings control chart used for internal quality control in human papillomavirus detection

**DOI:** 10.1186/s12985-022-01861-8

**Published:** 2022-07-28

**Authors:** Peiyi Peng, Xuehong Peng, Xiaoyang Jiao, Nuan Chen

**Affiliations:** 1 Department of Clinical Laboratory, The First Affiliated Hospital of Shantou University Medical College, Shantou, Guangdong China; 2 Shantou University Medical College, Shantou, Guangdong China; 3 Department of Thoracic Surgery, The First Affiliated Hospital of Shantou University Medical College, Shantou, Guangdong China

**Keywords:** Internal quality control, Levey–Jennings charts, HPV DNA detection

## Abstract

**Objective:**

The purpose of this study was to provide an updated estimate of the prevalences of different types of human papillomavirus (HPV) in females in Chaoshan District and to establish an internal quality control (IQC) method for excluding false-positive results in HPV detection by using the Levey–Jennings control chart.

**Method:**

HPV types were detected in 23,762 cervical samples by using PCR membrane hybridization. The means and standard deviations (SDs) of the positive rates were calculated, the Levey–Jennings chart was plotted, and the rules for “out of control” and “warning” were established. A set of standardized IQC for HPV DNA tests was developed based on the values and Levey–Jennings charts.

**Result:**

In 466 batches, the positive rate exceeded the 1 + 2SD rule 24 times, but there was no consecutive exceedance, which was considered “in control”. When the positive rate exceeded the 1 + 3SD rule 8 times with consecutive exceedance, it was considered “out of control”. Further examination revealed that detections showing “out of control” had an undesirable random error, indicating that contamination may occur due to improper operation.

**Conclusion:**

This unique Levey–Jennings control chart is a practical method for eliminating false-positive results in HPV DNA detection and should be widely applicable in molecular diagnostic laboratories.

## Background

Cervical cancer is the fourth most often diagnosed cancer in women and the fourth leading cause of cancer-related death [1]. HPV infection is the main cause of cervical cancer. HPV is a double-stranded DNA virus with icosahedral capsids that are not enclosed and can attack the cutaneous or mucosal epithelium [2]. To date, more than 100 distinct types of HPV have been identified, with approximately 20 types of HPV strains linked to cervical cancer. HPV DNA can be identified in virtually all cervical cancer tissues (> 99.7%) [3, 4], although HPV infection does not always cause cervical cancer. Based on their oncogenic potential, HPV types are divided into two risk classes: high-risk (HR-HPV) types are thought to be the aetiological agents of cervical cancer and other genital malignancies. Low-risk (LR-HPV) types are linked with benign genital warts [5]. HPV infection, particularly HR- HPV infection, has been linked to cervical squamous cell carcinoma [6, 7]. HPV infections may be temporary [8, 9]. However, HR-HPV persistent infection is a critical precursor for cervical cancer [10]. Notably, although all HR-HPV genotypes have the potential to cause cancer, some are more commonly associated with cancer than others. An increasing number of studies have been conducted to explore the prevalence of HPV and its genotype distribution in various geographical locations, with widely disparate results [11–14]. HPV infection rates and types vary by geography, according to an International Agency for Research on Cancer survey of healthy women. HPV-45 and 33 are the most common types in Africa, HPV-33 and 31 are the most common types in Europe, HPV-31, 33, and 45 are the most common types in the United States, and HPV-58 and 52 are the most common types in Asia [15]. These findings emphasize the significance of HPV genotyping in cervical cancer early diagnosis, therapy, and prognosis. In developed nations, cervical cancer incidence has decreased dramatically as a result of the widespread adoption of effective cervical cancer screening [16]. Women aged 30 to 65 should be examined with cytology and HPV testing (cotesting) every 5 years, according to the US Preventative Services Task Force (USPSTF) [17].

Currently, the most frequent procedures for cervical cancer screening include the Pap smear, the ThinPrep cytologic test (TCT), and HPV DNA testing [18]. The Pap smear, commonly known as the Pap test, is a screening procedure performed in the clinic to detect the presence of precancerous or cancerous cells on the cervix. However, Pap smears have progressively been superseded by TCT due to the limited sensitivity and high false-negative rate of the former, even though they are far less expensive [19]. TCT can only identify patients with aberrant cell morphology and cannot evaluate whether these individuals are at high risk of lesion development. TCT findings may also be impacted by specimen and slide quality, depending on the practitioner's expertise and experience [20]. The HPV DNA test is a suggested cervical cancer screening technique that not only can identify HPV but also can define the subtype. However, due to the great sensitivity of HPV DNA testing, there is a significant risk of false-positives [21], and false-positive results may arise due to the product carryover from earlier experiments or cross-contamination by positive samples during detection.

For cervical cancer screening, there is no well-organized training system or standardized cytology quality control (QC) criteria [22, 23]. IQC procedures must be created and conducted to ensure the accuracy of experimental results with the rising demand for HPV DNA testing for early cervical cancer screening. General approaches include the following: (a) ensuring that the experiment was accurate and that reagent blanks, positive quality control products, and negative quality control products were inserted randomly among clinical samples for each test to avoid false-positive results; (b) adding the detection reagent to the template DNA probe as an internal quality control. If there are no inhibition factors in the amplification reaction system and Internal Control (IC) points appear, the whole PCR amplification process can be monitored to avoid false-negative results. These measures have greatly improved the accuracy of measurement; however, the risk of a technician making a random error still exists.

Shewhart introduced the concept of control charts in 1924 and published it in 1931. He classified significant differences from standard quality as values higher than 3 SDs from the mean [24, 25]. Shewhart's control charts, according to Levey and Jennings, must be utilized in clinical laboratories to give a consistent way of assessing the reliability of the tests each run. For Levey–Jennings charts, the control limit is 3 SDs, as it is for Shewhart charts. In 1959, Henry explained how to use 1 SD, 2 SDs, or 3 SDs control limits, as well as the 95 percent confidence interval [26], while in 1977, Westgard et al. published clear rules for interpreting a control result that is 1 SD, 2 SDs, or 3 SDs from the mean [27, 28]. These charts use single-rule or multi-rule methods to specify the criteria for violations during data analysis, thereby reducing erroneous rejections while increasing actual error detection [29]. Many domestic and foreign researchers are currently using the Levey–Jennings control chart and the Westgard guidelines in practical research to investigate random and systematic errors in laboratory specimen testing [30]. Unfortunately, there are few reports on the use of Levey–Jennings charts in molecular diagnostics.

In this study, we aimed to provide an updated estimate of the type HPV prevalence in females in Chaoshan District, Guangdong Province, China, using PCR membrane hybridization measurements. Additionally, an IQC method was built based on the Levey–Jennings chart for monitoring HPV DNA testing, and the effects were confirmed by detection. The method including has two roles. The first role is to monitor short-term findings and confirm each batch of specimens, as well as to monitor the whole process to identify random and systemic errors. The second role is to use a control chart to graph successive quality control results, thereby providing an objective criterion of evaluation.

## Materials and methods

### Clinical samples

A total of 23,762 cervical cell samples were included in the study. The samples were collected from July 2019 to June 2021 at the First Affiliated Hospital of Shantou University Medical College. All methods in human-participant research were carried out in compliance with ethical guidelines and were approved by the Ethics Committee of the First Affiliated Hospital of Shantou University Medical College.

Sample collection: There was no vaginal cleaning, intravaginal medicine, or physical therapy for three days before the collection of the samples. There was no sexual activity within 24 h, and the women were not in their menstrual cycle when the samples were obtained.

### HPV DNA test

The HPV PCR membrane hybridization test (Hybribio Biotechnology Ltd. Corp, Guangdong Province, China) detects 15 different types of HR HPV (16, 18, 31, 33, 35, 39, 45, 51, 52, 53, 56, 58, 59, 66,68) [31] and 6 types of LR HPV (6, 11, 42, 43, 44, and CP8304) [32].

DNA extraction: Exfoliated cervical cells were obtained by centrifuging samples at 4 °C for 15 min at 13,000 rpm. The organic solvent extraction principle is applied to separate and precipitate the released DNA from the cell lysate. With the type test kit given by China Chaozhou Hybribio Biochemistry Co., Ltd., the PCR mix, Taq enzyme, and DNA template were combined in proportion. The following amplification conditions were used: 20 °C 10 min, 95 °C 9 min, 40 cycles (95 °C 20 s, 55° C 30 s, 72 °C 30 s), 72 °C 5 min, and 4 °C hold. In the hybridization procedure, all amplification products were adopted. The biotin, IC, and positive results showed distinct blue and purple dots after colour rendering. The positive genotypes were determined based on the distribution of HPV types in membrane strips. Throughout the procedure, negative, positive, and reagent blank controls were set up. Our hospital's HPV test participated in the national health and family planning commission's clinical test centre’s quality evaluation system, and all of them were qualified.

### Analysis of quality-control data for application to HPV DNA testing

The data were statistically analysed using the SPSS 25 and the Microsoft Excel Data Pack 2010. The quantile–quantile (Q–Q) plot was used to verify the normality of the distribution.

Two HPV subtypes with a high positive rate were selected for statistical quality control testing, and the positive rate of the HPV test for each batch was calculated. The positive rate of each batch was presented as ${\overline{\text{x}}}$, ${\overline{\text{x}}}$-1SD, ${\overline{\text{x}}}$ + 1SD, ${\overline{\text{x}}}$ + 2SD, and ${\overline{\text{x}}}$ + 3SD. IQCs of HPV DNA detection were set based on the Levey–Jennings map. Since the lowest positive rate was 0, only the upper part of the Levey–Jennings diagram was used for statistical quality control.

### IQC based on the Levey–Jennings chart is plotted and applied

The Levey–Jennings chart's x-axis represents the dates of 466 batches (7/1/2019–6/30/2021), in which ${\overline{\text{x}}}$, ${\overline{\text{x}}}$-1SD, ${\overline{\text{x}}}$ + 1SD, ${\overline{\text{x}}}$ + 2SD, and ${\overline{\text{x}}}$ + 3SD were designated on the y-axis. Based on the positive rates of HPV, each batch is calculated and plotted on the Levey–Jennings charts [25–28], the ${\overline{\text{x}}}$, ${\overline{\text{x}}}$-1SD, ${\overline{\text{x}}}$ + 1SD, ${\overline{\text{x}}}$ + 2SD, and ${\overline{\text{x}}}$ + 3SD values for the positive rates of HPV each batch were drawn on the y-axis of the Levey–Jennings chart to produce an IQC based on the Levey–Jennings chart for the positive rates of HPV each batch.

### Interpretation of control results

According to the Westgard‐Sigma rules [28, 33, 34], only if the negative quality controls are correct are the results considered to be out of control in the following situations: if the positive rate is more than 3 SDs above the mean (1–3S); if the positive rate is 2 SDs above the mean, which is a "warning " but not regarded as “out of control” (1–2S); or if two consecutive “warnings”have appeared (2 – 2S).

## Results

### Positive rate and genotype distribution of HPV

**Fig. 1:**
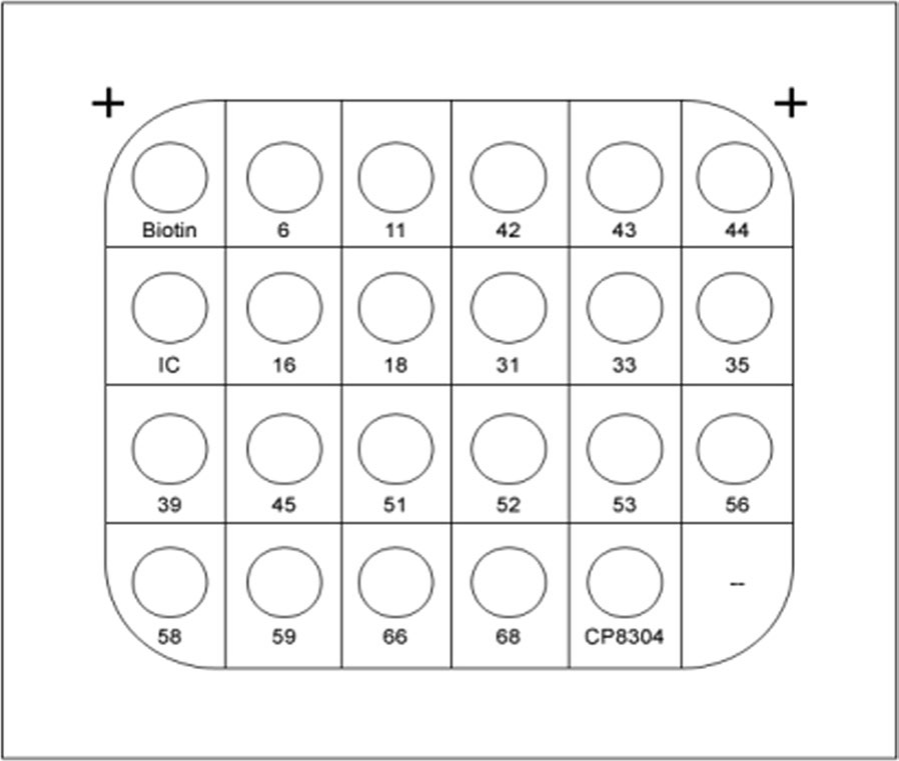
The distribution map of HPV types in membrane strips

**Fig. 2:**
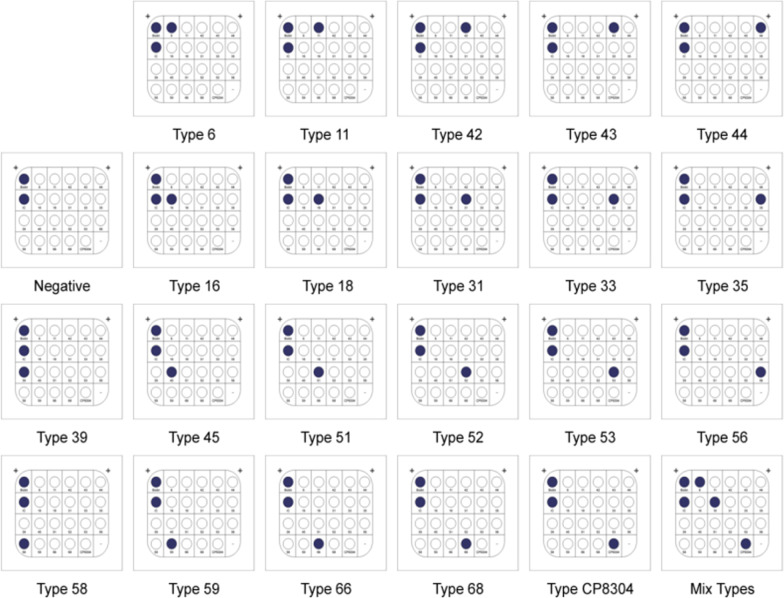
The HPV PCR membrane hybridization test results

**Fig. 3:**
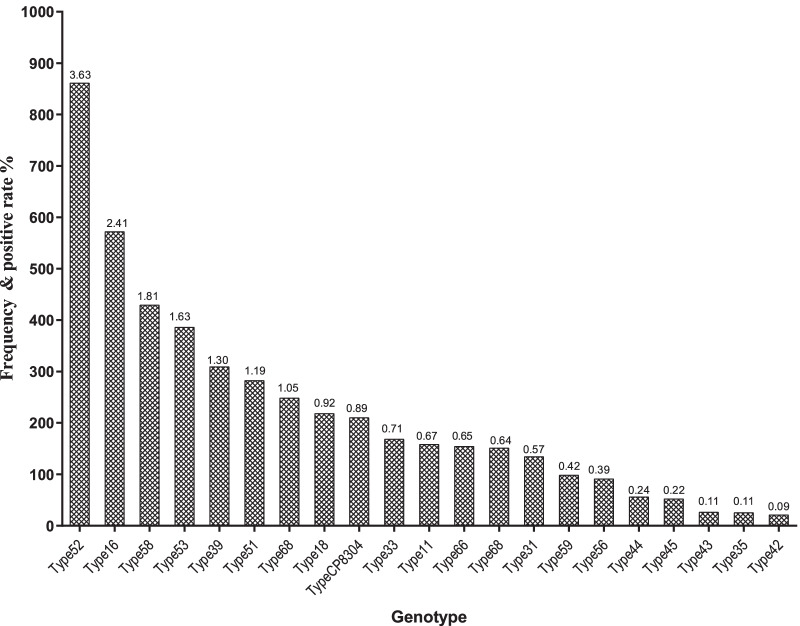
Frequency and prevalence of genotype of HPV among women

The total prevalence for HPV was 19.65% (4670/23762), and the 10 most prevalent genotypes of HPV were HPV 52 (862, 3.63%), HPV 16 (573, 2.41%), HPV 58 (430, 1.81%), HPV 53 (387, 1.63%), HPV 39 (310, 1.30%), HPV 51 (283, 1.19%), HPV 6 (249, 1.05%), HPV 18 (219, 0.92%), HPV CP8304 (211, 0.89%), and HPV 33 (169, 0.71%). (Table 1. *Frequency and positive rate of the HPV genotype among women)*. The HPV PCR membrane hybridization test results are presented in Fig. 1*The distribution map of HPV types in membrane strips,* Fig. 2*The HPV PCR membrane hybridization test results*, and Fig. 3*Frequency and prevalence of genotype of HPV among women.*

**Table 1:** Frequency and positive rate of the HPV genotype among women

HPV types	Frequency	Positive rate (%)
52	862	3.63
16	573	2.41
58	430	1.81
53	387	1.63
39	310	1.30
51	283	1.19
6	249	1.05
18	219	0.92
CP8304	211	0.89
33	169	0.71
11	159	0.67
66	155	0.65
68	152	0.64
31	135	0.57
59	99	0.42
56	92	0.39
44	57	0.24
45	53	0.22
43	27	0.11
35	26	0.11
42	22	0.09

### Acquisition of IQC datasets

**Fig. 4:**
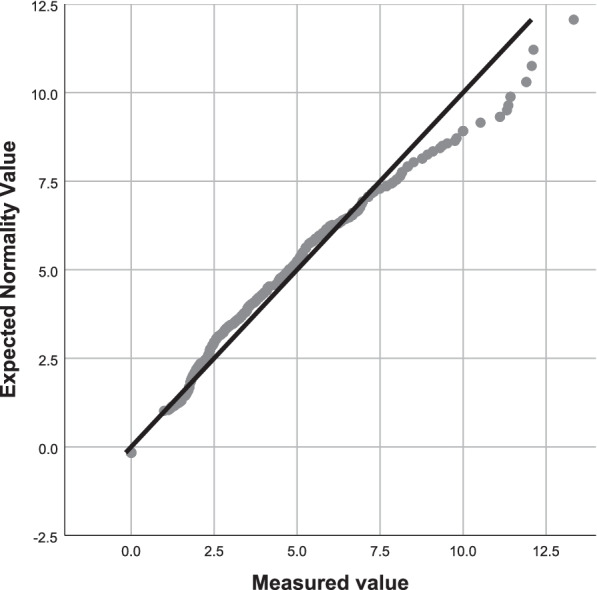
Q-Q Normality plot for positive rates of HPV 52

**Fig. 5:**
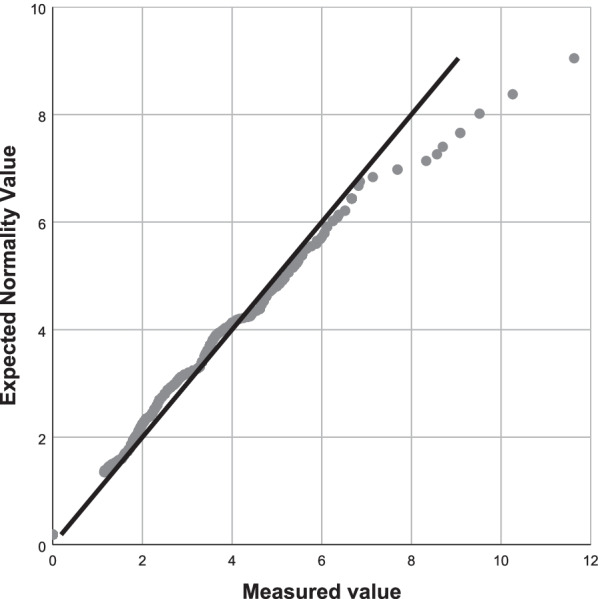
Q-Q normality plot for positive rates of HPV 16

The positive rates were highest in HPV 52 and 16. The positive rate in each batch was calculated. A normality test was performed on these data, and the results are shown in Fig. 4*Q–Q normality plot for positive rates of HPV 52* and Fig. 5*Q–Q normality plot for positive rates of HPV 16.* These data have been proven to be normally distributed.

### Creating the IQC based on the Levey–Jennings chart

**Fig. 6:**
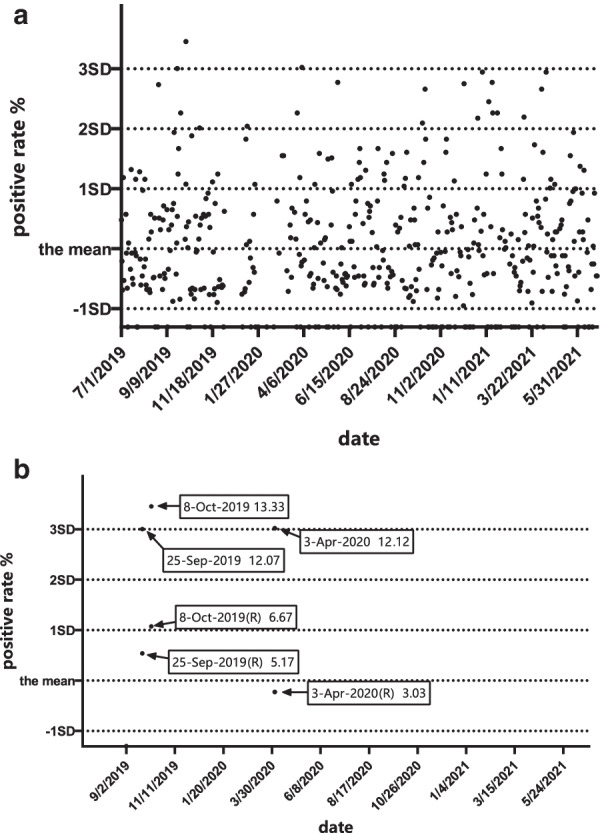
Levey–Jennings for positive rates of HPV 52

**Fig. 7:**
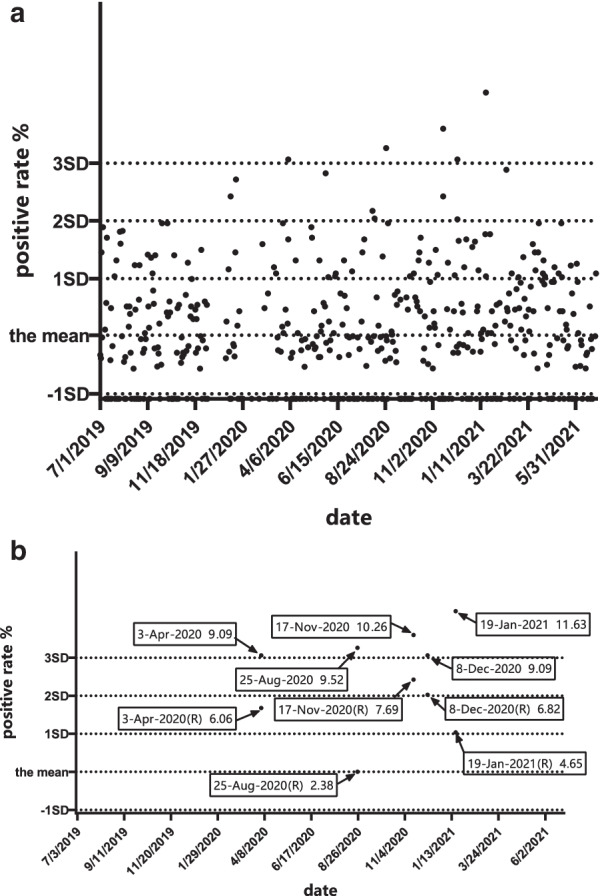
Levey–Jennings for positive rates of HPV 16

The mean positive rate of HPV 52 in the first 20 batches was 3.24%, and the SD was 2.20%, which is almost the same as 3.66% and 2.80% in all 466 batches; the two were not significantly different (*P* > 0.05). For HPV 16, the mean positive rate of the first 20 batches was 2.34%, and the SD was 2.29%, which is also almost the same as 2.38% and 2.19% in all 466 batches; these differences were not significant (*P* > 0.05). The results are presented in Table 2. *Comparison of the data of HPV 52 and 16 in the first 20 batches and all 466 batches*. Thus, in practical applications, the calculation can be carried out with 20 data points first, and then, the number of data points used for calculation can be adjusted at any time or regularly with the increase in detection times. Figure 6a *Levey–Jennings for positive rates of HPV 52* and Fig. 7a *Levey–Jennings for positive rates of HPV 16* were plotted based on these means and SDs.

**Table 2:** Comparison of the data of HPV 52 and 16 in the first 20 batches and all 466 batches

HPV type	52	16
Frequency	862	573
Batches	466	466
Means of the positive rates	3.66%	2.38%
SD	2.80%	2.19%
Means of the first 20 batches positive rates	3.24%	2.34%
SD	2.20%	2.29%
t-test, *P*-values	0.409 > 0.05	0.942 > 0.05

From Fig. 6a, the positive rate exceeded the 1 + 2SD rule 18 times, but there was no consecutive exceedance in the 466 batches. The positive rate exceeded the 1 + 3SD rule 3 times. Therefore, according to the rule, 3 results were out of control in 466 batches (September 25 and October 8, 2019, and April 3, 2020). Overall, the frequency of “out of control” runs was normally distributed.

From Fig. 7a, the positive rate exceeded the 1 + 2SD rule 6 times, but there was no consecutive exceedance in the 466 assays. The positive rate exceeded the 1 + 3SD rule 5 times. Consequently, 5 results that were out of control were observed in 466 batches (April 3, August 25, November 17, and December 8, 2020, and January 19, 2021).

Because the amount of data is too large, we give priority to data representing “out of control”and attempted to demonstrate “ out of control”and “in control”in the QC chart at the same time. We deleted some of the data that only represented had “in control”. Accordingly, only HPV 52 test data from September 25, October 8, 2019, and April 3, 2020, were left. Figure 6b *Levey–Jennings for positive rates of HPV 52* showed that the positive rate of test results exceeded 3 SD and decreased significantly upon retesting; these results were considered“in control”.Similarly, when other data were removed and only the test data for HPV 16 on April 3, August 25, November 17, December 8, and January 19, 2021, were left, the positive rate of test results exceeding 3SD also decreased after retesting, which was considered“in control” and is shown in Fig. 7b *Levey–Jennings for positive rates of HPV 16*.

## Discussion

In this study, the prevalence for HPV was 19.65%, and HPV 52 (3.63%), 16 (2.41%), 58 (1.81%), 53 (1.63%), and 39 (1.30%) were the most common HPV genotypes in the Chaoshan District, Guangdong Province. The prevalence of HPV infection in Beijing is 19.1%, with the most frequent genotypes being HPV 52, HPV 16, and HPV 58 [35]. The prevalence of HPV infection in Shenzhen is 15.9%, with the most frequent genotypes being HPV 52, HPV 16, and HPV 53 [36]. Our results were consistent with the results of other areas in China.

In recent years, local and international research has focused on the necessity of quality control in molecular diagnostics [37–41]. The use of routine test results for IQC has been used in HPV-DNA testing. It is important to plot a quality control chart based on the means of the daily assay data to determine whether the test result is out of control. The results obtained from the qualitative PCR method are categorical data that have two types of results: negative and positive. The daily positive rate can be calculated as measurement data for IQC. After analysing the positive rate of the HPV DNA results, a normal distribution, which is one of the characteristics of these results, has been found. In clinical practice, 20 batches of tests can be carried out first, and then, the quality control chart can be drawn according to the results of the 20 batches of tests. This method was tested in our previous works, which included 23,762 samples and 466 batches of tests. The positive rates of 466 batches presented with a normal distribution. However, if the results are not normally distributed, this method cannot be applied effectively. We analysed the mean positive rate of the first 20 batches and the mean positive rate of the 466 batches with the t-test, and no significant difference was found (*P* > 0.05); *therefore, a quality control chart such as the Levey–Jennings plot could be built. The SD is often used to verify systematic and random errors, and according to the rejection rules for the computed decision ranges, generally, 1 SD, 2 SDs, and 3 SDs from the mean of the* positive rate are used in the first 20 batches.[42]. As this method is only used for false-positive laboratory quality control, the results above the means are the only consideration. Thus, only the top half of the original Levey–Jennings quality control chart is presented in this method.

One of the “out of control” manifestations that exist in this positive rate monitoring internal quality control method is the curve drift up. This change may be abrupt, indicating a random error. Any data point that is outside of the 3 SDs ranges is considered an undesirable random error, indicating that contamination may occur due to improper operation, such as specimen leakage, positive quality control product leakage, standard leakage, or cross-contamination between samples. This indicates the possibility of a false-positive result. In our study, according to Figs. 6 and 7, it was obvious that these outliers of allowable range experiments were most likely random errors caused by a technician during the sample's processing. All of these may have potential pollution and should be analysed to determine whether there are false-positives caused by cross-contamination between samples due to improper operation. It is worth mentioning that the experimenters had already retested the samples whose results were out of control on the day of the experiment, and the results show that all of the outside of allowable ranges results are indeed caused by cross-contamination between samples. This provides further evidence of the feasibility of this method and shows one of the advantages of this method, which is helping the experimenters make a judgement of the result more objectively and reduce errors that are caused by inexperience.

The second is the upwards trend of the curve; this change may be gradual, which indicates the probability of cumulative product contamination. The laboratory amplification products gradually accumulate and thus increase the positive rate of the result, indicating a systematic error. Under this circumstance, the laboratory environment and instruments need to be thoroughly cleaned.

The positive rate of the result is also influenced by many other factors, so, full consideration is needed when analysing the “out of control” results. In a specific clinical PCR laboratory, the positive rate would not change much because the people they serve usually stay the same or do not change much. Therefore, when the positive rate rises to a very high level in one day, for example, up to 15%, or the daily positive rate increases gradually, this case needs to be carefully studied due to the possibility of false-positives. Additional factors, including pipette tip contamination, aerosol contamination, and cross-contamination of prepositive samples before sending them out must be carefully checked. Contamination can result in false-positive test findings, which can have serious clinical repercussions. A method indicating the analytical series rejection guidelines, as well as quick tag flagging of a rejection event, should be implemented in each laboratory. In practice, a Lever-Jennings quality control chart can be constructed based upon the daily positive rate in a laboratory, which can be incorporated into nucleic acid detection. This unique Levey–Jennings control chart addresses the issue of insufficient IQC, and should be extensively applicable in molecular diagnostic laboratories.

## Data Availability

Not applicable.
